# The phenotype of Floating-Harbor syndrome: clinical characterization of 52 individuals with mutations in exon 34 of *SRCAP*

**DOI:** 10.1186/1750-1172-8-63

**Published:** 2013-04-27

**Authors:** Sarah M Nikkel, Andrew Dauber, Sonja de Munnik, Meghan Connolly, Rebecca L Hood, Oana Caluseriu, Jane Hurst, Usha Kini, Malgorzata J M Nowaczyk, Alexandra Afenjar, Beate Albrecht, Judith E Allanson, Paolo Balestri, Tawfeg Ben-Omran, Francesco Brancati, Isabel Cordeiro, Bruna Santos da Cunha, Louisa A Delaney, Anne Destrée, David Fitzpatrick, Francesca Forzano, Neeti Ghali, Greta Gillies, Katerina Harwood, Yvonne M C Hendriks, Delphine Héron, Alexander Hoischen, Engela Magdalena Honey, Lies H Hoefsloot, Jennifer Ibrahim, Claire M Jacob, Sarina G Kant, Chong Ae Kim, Edwin P Kirk, Nine V A M Knoers, Didier Lacombe, Chung Lee, Ivan F M Lo, Luiza S Lucas, Francesca Mari, Veronica Mericq, Jukka S Moilanen, Sanne Traasdahl Møller, Stephanie Moortgat, Daniela T Pilz, Kate Pope, Susan Price, Alessandra Renieri, Joaquim Sá, Jeroen Schoots, Elizabeth L Silveira, Marleen E H Simon, Anne Slavotinek, I Karen Temple, Ineke van der Burgt, Bert B A de Vries, James D Weisfeld-Adams, Margo L Whiteford, Dagmar Wierczorek, Jan M Wit, Connie Fung On Yee, Chandree L Beaulieu, FORGE Canada Consortium, Sue M White, Dennis E Bulman, Ernie Bongers, Han Brunner, Murray Feingold, Kym M Boycott

**Affiliations:** 1 Children’s Hospital of Eastern Ontario, University of Ottawa, Ottawa, ON, K1H 8L1, Canada; 2 Division of Endocrinology, Boston Children's Hospital, Boston, USA; 3 Department of Human Genetics, Radboud University Nijmegen Medical Centre, Nijmegen, the Netherlands; 4 Division of Genetics, Boston Children’s Hospital and The Manton Center for Orphan Disease Research at Children’s Hospital, Boston, USA; 5 Children’s Hospital of Eastern Ontario Research Institute, University of Ottawa, Ottawa, ON, Canada; 6 Department of Medical Genetics, University of Alberta, Edmonton, Canada; 7 NE Thames Genetics Service, Great Ormond Street Hospital NHS Foundation Trust, Great Ormond Street, London, UK; 8 Oxford University Hospitals NHS Trust, Oxford, UK; 9 McMaster University Medical Centre, Hamilton, Canada; 10 Service de Neuropédiatrie AP-HP, Centre de Référence des anomalies de développement et syndromes malformatifs, Hôpital Armand Trousseau, Paris, France; 11 Institut für Humangenetik, Universitaetsklinikum Essen, Essen, Germany; 12 Department of Molecular Biology University of Siena, Medical Genetics Unit, Siena, Italy; 13 Department of Paediatrics, Hamad Medical Corporation, Clinical and Metabolic Genetics, Doha, Qatar; 14 Policlinico Tor Vergata University Hospital, Rome, Italy; 15 Department of Medical, Oral and Technological Sciences, D’Annunzio University, Chieti, Italy; 16 Hospital de Santa Maria Lisboa, Lisbon, Portugal; 17 Faculdade de Medicina da Pontifícia Universidade Católica do Rio Grande do Sul, Rio Grande do Sul, Brazil; 18 Centre de Génétique Humaine, Institut de Pathologie et de Génétique, Charleroi, B-6041, Belgium; 19 MRC Human Genetics Unit MRC IGMM, University of Edinburgh Western General Hospital, Crewe Road, Edinburgh, Scotland; 20 Medical Genetics Unit, Galliera Hospital, Genova, Italy; 21 North West Thames Regional Genetics Service, Northwick Park Hospital, Harrow, UK; 22 Bruce Lefroy Centre, Murdoch Childrens Research Institute, Royal Children’s Hospital, Melbourne, Australia; 23 Division of Pediatric Endocrinology, St. Joseph's Children's Hospital, Paterson, NJ, USA; 24 Department of Clinical Genetics, Free University Medical Center, Amsterdam, the Netherlands; 25 Département de Génétique et Cytogénétique, Centre de Référence “Déficiences intellectuelles de causes rares”, University Paris 6, upmc, Groupe Hospitalier Pitié-Salpêtrière, Paris, France; 26 Department of Human Genetics, Nijmegen Centre for Molecular Life Sciences, Institute for Genetic and Metabolic Disease, Radboud University Nijmegen Medical Centre, Nijmegen, the Netherlands; 27 Department Genetics, University of Pretoria, Pretoria, South Africa; 28 Genetics Division, St. Joseph’s Children’s Hospital, Paterson, NJ, USA; 29 Service Neurogenetique, Hopital Pitie Salpetriere, Paris, France; 30 Department of Clinical Genetics, Center for Human and Clinical genetics (CHKG), Leiden University Medical Center, Leiden, The Netherlands; 31 Unidade de Genética, Instituto da Criança, Hospital das Clínicas-Faculdade de Medicina Universidade de São Paulo, São Paulo, Brazil; 32 Department of Medical Genetics, Sydney Children's Hospital, Sydney, Australia; 33 Department of Medical Genetics, University Medical Center Utrecht, Utrecht, the Netherlands; 34 Laboratoire Maladies Rares: Génétique et Métabolisme, Service de Génétique Médicale, Centre Hospitalier Universitaire de Bordeaux, University of Bordeaux, Bordeaux, France; 35 Division of Genetics, Department of Pediatrics, University of California, San Francisco, California, USA; 36 Department of Health, Clinical Genetic Service, HKSAR, China; 37 Institute of Maternal and Child Research, Faculty of Medicine, University of Chile, Santiago, Chile; 38 Department of Clinical Genetics, Oulu University Hospital and University of Oulu, Oulu, Finland; 39 Department of Clinical Genetics, Odense University Hospital, Odense C, Denmark; 40 Institute of Medical Genetics, University Hospital of Wales, Cardiff, CF14 3PY, UK; 41 Northampton General Hospital Trust, Northampton, UK; 42 Serviço de Genética Médica, Centro Hospitalar e Universitário de Coimbra, Coimbra, Portugal; 43 Ambulatório de Genética Médica da Prefeitura de Porto Alegre, Porto Alegre, Brazil; 44 Department of Clinical Genetics, Erasmus MC Rotterdam, Rotterdam, The Netherlands; 45 Human Development and Health, Faculty of Medicine, University of Southampton, Wessex Clinical Genetics Service, Princess Anne Hospital, Southampton, UK; 46 Department of Genetics and Genomic Sciences, Mount Sinai School of Medicine, New York, NY, USA; 47 Department of Clinical Genetics, Level 2A, Laboratory Medicine Building, Southern General Hospital, 1345 Govan Rd, Glasgow, Scotland, G51 4TF, UK; 48 Department of Pediatrics, Leiden University Medical Center, Leiden, The Netherlands; 49 Genetic Health Services Victoria, Murdoch Children's Research Institute, Royal Children's Hospital, Melbourne, Victoria, Australia; 50 Division of Genetics, Boston Children’s Hospital, Boston, USA

**Keywords:** *SRCAP*, Floating Harbor syndrome, Phenotype, Short stature

## Abstract

**Background:**

Floating-Harbor syndrome (FHS) is a rare condition characterized by short stature, delays in expressive language, and a distinctive facial appearance. Recently, heterozygous truncating mutations in *SRCAP* were determined to be disease-causing. With the availability of a DNA based confirmatory test, we set forth to define the clinical features of this syndrome.

**Methods and results:**

Clinical information on fifty-two individuals with *SRCAP* mutations was collected using standardized questionnaires. Twenty-four males and twenty-eight females were studied with ages ranging from 2 to 52 years. The facial phenotype and expressive language impairments were defining features within the group. Height measurements were typically between minus two and minus four standard deviations, with occipitofrontal circumferences usually within the average range. Thirty-three of the subjects (63%) had at least one major anomaly requiring medical intervention. We did not observe any specific phenotype-genotype correlations.

**Conclusions:**

This large cohort of individuals with molecularly confirmed FHS has allowed us to better delineate the clinical features of this rare but classic genetic syndrome, thereby facilitating the development of management protocols.

## Background

Floating-Harbor syndrome (FHS [MIM 136140]) is a rare disorder characterized by short stature with delayed bone age, deficits in expressive language and a distinctive facial appearance. The name of the syndrome is derived from the two hospitals where the first patients were reported over 35 years ago [1,2]. Recently, we used exome sequencing to investigate a cohort of 13 unrelated individuals with classic features of FHS and identified heterozygous mutations in *SRCAP* [MIM 611421] as causative of this disorder [3]. All reported mutations were truncating and occurred between codons 2,407 and 2,517 in exon 34 resulting in loss of three C-terminal AT-hook motifs. *SRCAP* encodes a SNF2-related chromatin-remodeling ATPase that serves as a coactivator for CREB-binding protein, better known as CBP, the major cause of Rubinstein-Taybi syndrome (RTS). The disrupted interaction between these two proteins likely explains some of the clinical overlap between FHS and RTS [4]. The mechanism of disease in FHS is suspected to be dominant-negative [3] due to the non-random clustering of truncating mutations in the final exon that result in the loss of the major transactivation function of SRCAP located in a 655 residue C-terminal fragment, evidence that expression of a construct solely consisting of the CBP interaction domain of SRCAP strongly inhibits CREB-mediated transactivation in a dominant-negative fashion [5], and the existence of patients with haploinsufficiency of *SRCAP* who do not have features of FHS [3].

Many of the features of FHS are non-specific (short stature, delayed bone age, and language delays) and if the distinctive facial features are not recognized, this diagnosis can be difficult. Several years ago, Feingold [6] provided a thirty-two year follow-up on the first reported patient accompanied by a review of the literature. He suggested that some of the patients reported to have FHS did not fit the classical description and likely had a different condition. With the availability of a molecular test, we are now able to further delineate the distinctive and recognizable features of this syndrome.

## Methods

### Subjects and clinical data

Individuals with a presumptive clinical diagnosis of FHS were invited to be part of this study. Clinical data was collated from three sources: FORGE Canada Consortium (Finding of Rare Disease Genes in Canada), based at the Children’s Hospital of Eastern Ontario, the Manton Center for Orphan Disease Research at Boston Children’s Hospital, and the Radboud University Nijmegen Medical Centre. All samples that were referred for analysis were accepted for the study. Approval of the study design was in compliance with the Helsinki Declaration and was obtained from each of the participating institutions’ research boards. Free and informed consent was obtained from each study subject (or guardian, if appropriate) prior to enrollment. Recruitment e-mails were sent to all members of the Floating-Harbor syndrome support group. Interested families or physicians contacted the genetic counselor at the Manton Center. A medical history questionnaire was administered to the family or physician via telephone, which reviewed all pertinent medical and developmental history, as well as FHS-specific questions (*see* Additional file 1). Referring providers who submitted cases directly to the above institutions completed the same questionnaire. In most cases, clinical photographs were available prior to molecular testing and the likelihood of finding a mutation was noted. Due to the diversity of the sample sources, there was wide pre-test probability of referred individuals actually having FHS, as this is a rare condition and most clinicians do not have familiarity with it. The clinical information from the first 13 subjects described by Hood et al. [3] was also included.

### Molecular analysis

Sanger sequencing of exons 31–34 of *SRCAP* was performed using DNA samples from individuals with suspected FHS (*see* Additional file 2). When available, parental studies were performed to determine *de novo* or inherited status. The clinical information, from twenty-seven individuals who did not carry a mutation in exons 31–34 of *SRCAP*, was used to help clarify key diagnostic features. For three individuals, who most closely resembled the FHS phenotype and for which no mutations were identified in exon 34, complete sequencing of the *SRCAP* gene was performed (primer sequences available on request).

## Results and discussion

### Molecular

In total, 24 males and 28 females were identified with mutations in *SRCAP*; 39 new individuals and 13 previously reported [3]. Ages at time of data collection ranged from two years to 52 years of age. The average age of diagnosis was 8 years. Two mother/daughter pairs [7,8] and a number of the other subjects have been previously reported in the literature [1,3,6,7,9-11]. All the mutations identified in our cohort were truncating (nonsense or frameshift) alleles (Table 1). Two mutations are recurrent; the Arg2444* mutation was observed in about half (24/52) (including the original patient described by Pelletier and Feingold [1]), while the Arg2435* mutation was present in approximately one quarter (13/52) of the individuals with FHS. In our original cohort of 13 patients with FHS we delineated the boundaries of the critical region to between codons 2407 and 2517. The extended cohort of molecularly-defined patients we present here extends the critical region to between codons 2389 and 2748, a further 249 amino acids in exon 34. Interestingly, the boundaries of this critical region are delineated by mutations observed in our two mother-daughter pairs (Table 1), however, the significance of this finding is unclear.

**Table 1:** **Mutations detected in exon 34 of *SRCAP *in individuals with FHS**

	**c. DNA**	**Frequency (52)**	**Comments**
Glu2389*	c.7165G > T	2	Mother/Daughter [8]
Gln2407*	c.7219C > T	1	
Gln2407fs*35	c.7218_7219delTC	1	
Asn2410fs*32	c.7230insA	1	
Thr2425fs*17	c.7274insC	1	
Arg2435*	c.7303C > T	13	2nd Recurrent mutation
Ala2440fs*3	c.7316dupC	1	
Arg2444*	c.7330C > T	24	Most frequent Recurrent mutation
Pro2459fs*125	c.7374dupT	2	
Pro2459fs*16	c.7376delC	1	
Thr2512fs*5	c.7533_7534insAA	1	
Gln2517fs*5	c.7549delC	1	
Asn2618fs*11	c.7852insC	1	
Arg2748*	c.8242C > T	2	Mother/Daughter [7]

### Facial gestalt

**Figure 1:**
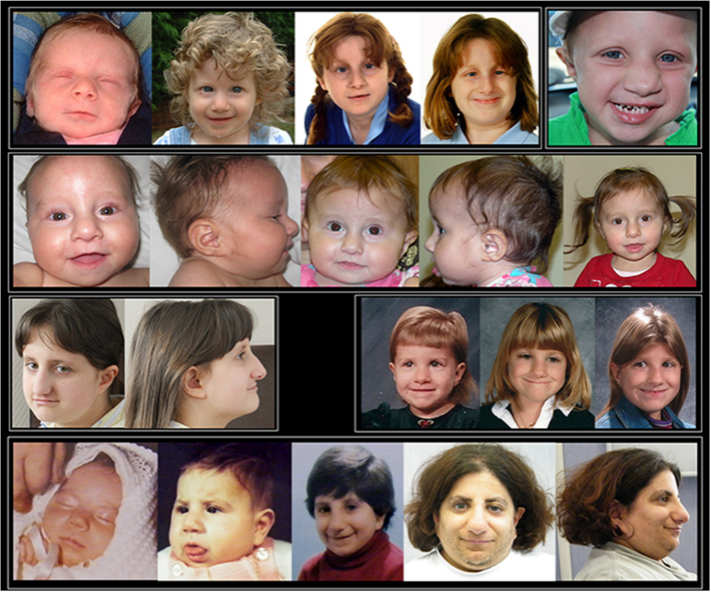
Facial photographs of 6 females with FHS with the common Arg2444* mutation.

**Figure 2:**
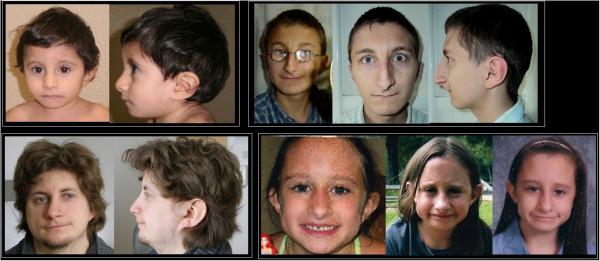
Facial photographs of 4 individuals with FHS of varying ages with the Arg2435* mutation.

**Figure 3:**
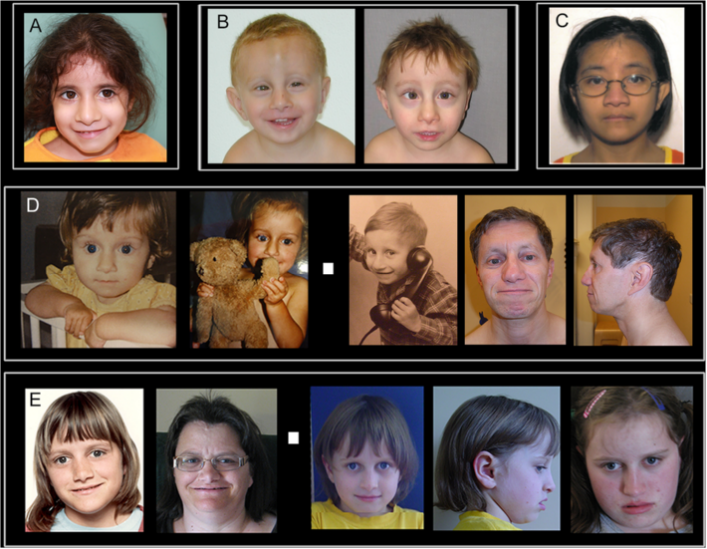
**Facial photographs of 7 individuals with FHS as examples of the other mutations. A**. A female with the Gln2407* mutation. **B**. A male with the Ala2440fs*3 mutation. **C**. A female with the Asn2618fs*11 mutation. **D**. A female and male with the Pro2459fs*125 mutation. **E**. A mother and daughter with the Arg2748* mutation.

The face of FHS is the most distinctive aspect of this syndrome (Figures 1, 2 and 3), and although there are changes with age, the cardinal features, as originally described [1,2,4], remain constant. The overall facial shape is triangular. The nose is narrow at the root and broadens to the tip. The columella is low hanging, nares are large and the philtrum is often short. The upper vermillion is typically thin and the lower lip is often everted. The lips tend to be in a horizontal plane at rest or when smiling. The eyes are frequently deep set and the eyelashes tend to be long. The ears can be low set and large in appearance. As seen in the photos, the FHS phenotype is more difficult to recognize in infancy.

### Skeletal

**Figure 4:**
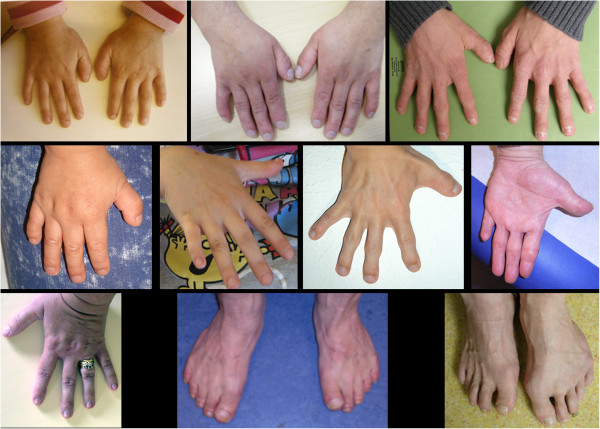
**Hands and feet of individuals with FHS.** Clinical photos demonstrating the variability of features ranging from unremarkable to brachydactyly, short broad thumbs and big toes, broad fingertips.

Of the 17 individuals where thumb morphology was formally assessed, broad thumbs were only seen in 10 individuals indicating that they are a frequent but not mandatory finding in FHS. The differential diagnosis of broad thumbs includes Rubinstein-Taybi syndrome, where they are a cardinal feature. FHS is also in the differential, which is logical as SRCAP interacts with CBP. Other skeletal findings include broad first toes and brachydactyly. Broad fingertips are seen frequently, and the fingers are often described as being clubbed, although would be more accurately classified as having broad fingertips (Figure 4). Leisti et al. [2] reported a right-sided pseudoarthrosis-type anomaly of the clavicle noted at age two in one of their patients. Four individuals in our series have uni- or bilateral clavicular anomalies including pseudoarthroses or hypoplasia. Two individuals have 11 pairs of ribs and four have hip dysplasia.

### Growth

**Figure 5:**
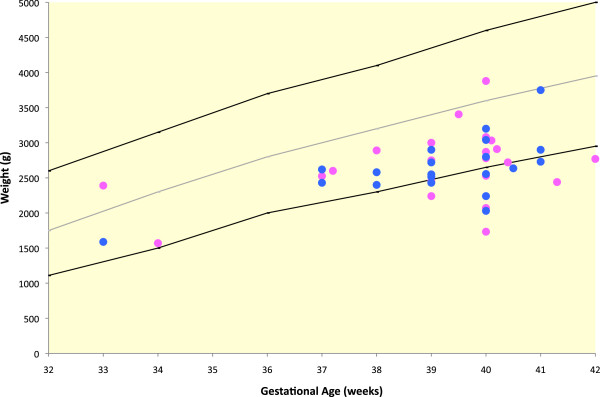
**Birth weights of individuals with FHS.** Male birth weights - blue dots; Female birth weights – pink dots. The mean, 5th and 95th confidence intervals are indicated.

**Figure 6:**
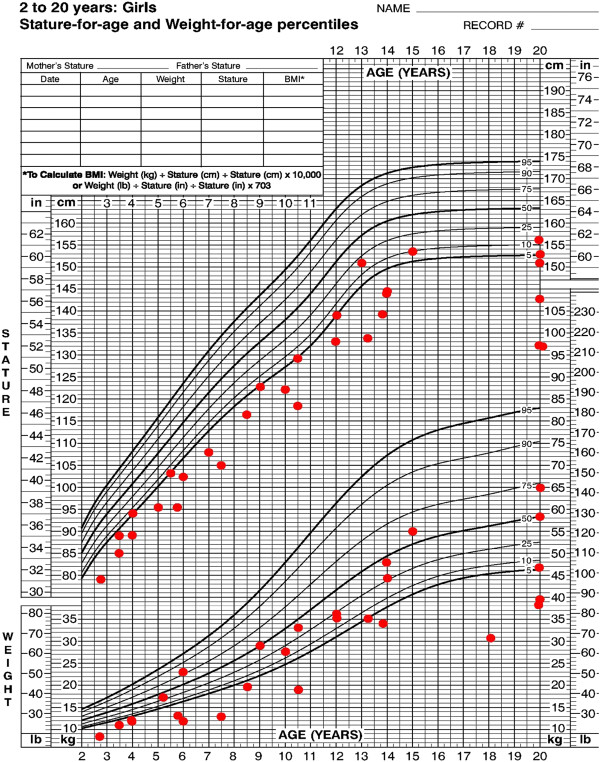
**Height and weight of female individuals with FHS.** Each point represents a single individual’s measurements at the time of data collection.

**Figure 7:**
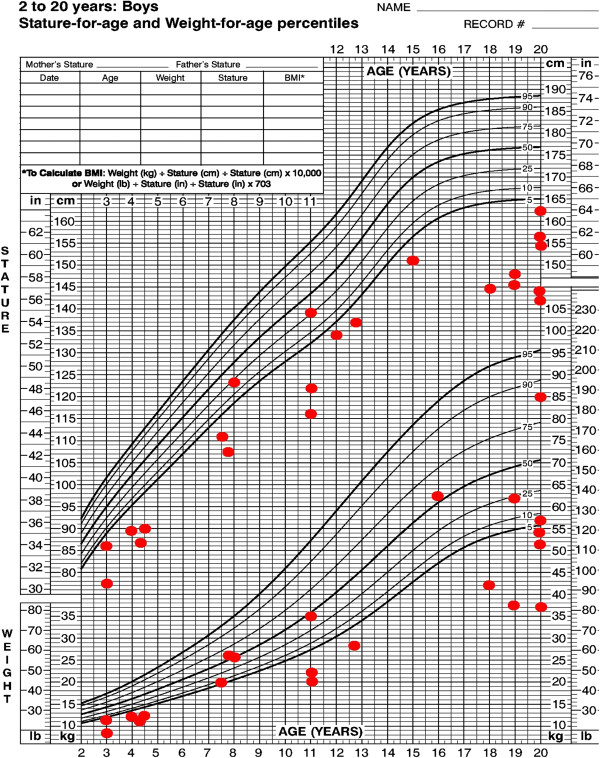
**Height and weight of male individuals with FHS.** Each point represents a single individual’s measurements at the time of data collection.

**Figure 8:**
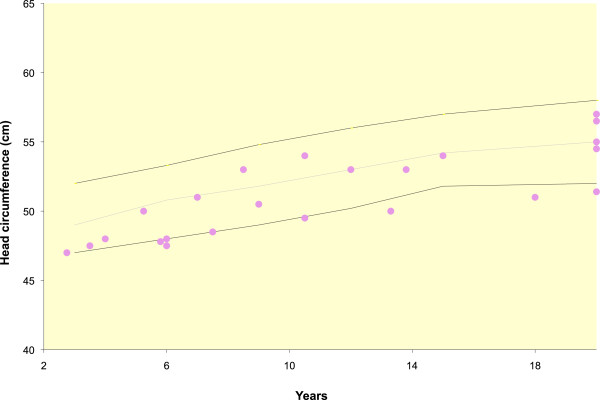
**OFCs of females with FHS.** Each point represents a single individual’s measurements at the time of data collection. The mean, 5th and 95th confidence intervals are indicated.

**Figure 9:**
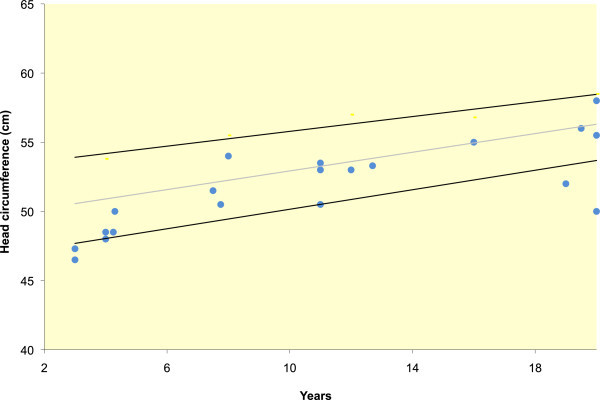
**OFCs of males with FHS.** Each point represents a single individual’s measurements at the time of data collection. The mean, 5th and 95th confidence intervals are indicated.

Where available, growth parameters were plotted on aggregate graphs. Thirteen of 49 individuals had birth weights less than the third percentile (Figure 5). For females, the maximum height was at the 20th percentile, with most data points between minus two and minus four standard deviations (SDs) (Figure 6). For the males, the height measurements varied more widely, with maximum height at the 25th percentile and two adult heights below four SDs (Figure 7). Occipito-frontal circumferences (OFC) were more variable, with most being well within the average range (Figures 8 &9). Seven individuals had OFCs less than two standard deviations, and only one measurement was less than minus 3 SDs. This suggests relative sparing of head size in relation to stature. Body weights were not consistent to suggest a particular body habitus for this syndrome, and probably reflect the variability seen in the general population.

### Bone age and endocrine

**Figure 10:**
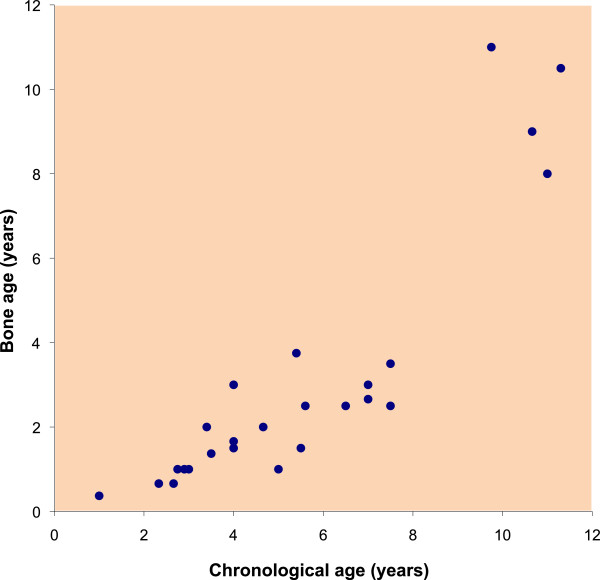
Bone age values plotted against chronological age for 25 individuals with FHS.

Bone age values were plotted against chronological age (Figure 10) and all values in subjects less than 8 years old showed significant delays. There were no data values between ages 8–10 years, however, the bone ages approached the chronological age or became advanced after age 10 years. A number of participants in this study have been on growth hormone (GH) therapy, which may alter the natural history of growth in this population. Two of our subjects have been assessed in more detail regarding this issue [9,10]. Some GH treated individuals with FSH had documented GH deficiency, while others had modest responses to treatment despite normal levels of GH [9,10,12]. Early puberty has previously been reported [13] in FHS and was documented in four individuals in our study. Some of our subjects are currently pre-pubertal, while others could not accurately report pubertal timing, rendering the data incomplete. However, early puberty could explain the advanced bone age seen in teenage individuals with FHS as well as contributing to shorter adult heights.

### Structural anomalies

A number of structural anomalies were detected in our cohort (Table 2), but no particular finding was seen with enough frequency to consider it a distinguishing feature of this syndrome. However, as some anomalies may affect clinical management, comprehensive screening is necessary in this population.

**Table 2:** Frequency of different clinical features in individuals with FHS

**Clinical feature**	**Frequency reported**
Eyes	
• Strabismus	7/43
• Hyperopia	5/43
• Nystagmus	1/43
Ears	
• Recurrent otitis media/T-tube placement	6/52
• Hearing loss	9/52
• Cochlear anomaly	1/U
Other ENT	
• Cleft lip and pseudocleft lip	2/52
• Velopharyngeal insufficiency	2/U
• Choanal atresia	1/U
Dental Issues	
• Small teeth/increased spacing	13/38
• Cavities	6/38
• Malocclusion/underbite	3/38
Cardiac Malformation*	3/52
Gastrointestinal	
• Motility issues (reflux/constipation)	13/52
• Colonic stricture	1/U
• Celiac disease	2/52
Genitourinary	
• Cryptorchidism	5/24
• Renal/collecting system anomalies	7/U
Seizures	6/52
Hypothyroidism	2/52

U – denominator unknown.

* Mild aortic coarctation, atrial septal defect, Tetrology of Fallot.

### Voice quality and language

A high-pitched voice is often commented upon in individuals with FHS and was reported in 8/11 individuals. Others noted a nasal quality to the voice. An additional individual had documented velopharyngeal insufficiency (VPI), which may indicate that VPI is under-recognized. Expressive language delay is a cardinal feature of this syndrome, and was reported in all subjects. There was significant variability in severity with one individual who was bilingual, while another could only speak a few words as an adult. However, language development could potentially be hampered by the high frequency of recurrent otitis media and conductive hearing loss found in our cohort.

### Cognition

The cognitive abilities in individuals with FHS range from average (IQ of 104) to significant intellectual impairment in a few instances. Most individuals had some modifications of their schooling (37/41). Obtaining full psychoeducational assessments on this cohort was beyond the scope of this study. However, when assessing global cognition in an individual with FHS, one must consider the language impairments, and in some instances sensory impairments, and adjust accordingly.

### Behaviour

The caregivers, in comparison to physicians, who filled out the questionnaires, often commented upon behavioral issues for their children (5/25). It is likely that these issues are under-recognized in this population. Rigid mannerisms were observed (7/25), as were some obsessive tendencies (e.g. skin picking). Parents often described their children as anxious individuals and attention deficit hyperactivity disorder (ADD or ADHD) was common (9/32).

We acknowledge that the data collection in our study was incomplete as data was obtained from a number of sources without a centralized clinical assessment. We also recognize that the ethnic backgrounds of the study subjects were mostly Caucasian and that FHS may be more difficult to diagnosis in other populations. However, three individuals of Chinese origin were clinically diagnosed and identified to have mutations in *SRCAP*. In addition to growth and developmental issues, all of these subjects had classical FHS facial features, which were distinct from those of their family members.

Lastly, we evaluated for the presence of a genotype-phenotype correlation in FHS. Upon review of the clinical data, no clinical features were identified which discriminated between the different mutations. Given that all mutations cause truncation in a very defined area of the gene, this observation was not entirely unexpected.

### Development of diagnostic criteria

The indication for analysis of the *SRCAP* gene was a presumptive diagnosis of FHS. The majority of those who underwent testing had short stature, delayed bone age, language delays and a distinctive facial appearance, usually with a prominent nose. Clinicians very familiar with FHS were able to distinguish those who ultimately carried a mutation in *SRCAP,* by his/her clinical information and facial photographs, from those who did not have a mutation. Those individuals who were referred who did not have a mutation detected often had dysmorphic facial features, but these were distinct from the classical FHS gestalt, making facial features the defining characteristic of FHS. The nose is quite distinctive in FHS with its overall triangular appearance, the orientation and size of the nares and the low hanging columella. The linear orientation of the mouth, at rest or when smiling, is also an important defining feature. Additional consistent features of those who tested negative were a formal diagnosis of autism or head circumferences at a comparatively smaller OFC percentile than that for height. Russell-Silver syndrome and 3-M syndrome are included in the differential diagnosis for FHS, but we do not believe any of the patients in our negative group had either of these diagnoses.

Three individuals, whose phenotype most closely resembled FHS, had sequencing of the entire *SRCAP* gene to explore the possibility of mutations outside of exons 31–34. However, no mutations were detected. It is plausible that their phenotypes could be due to a mutation in another gene that codes for a protein, which interacts with SRCAP and CBP*.* Further research is needed to elucidate this possibility. Given that we have no evidence of genetic heterogeneity within our cohort, we conclude that the detection of a truncating mutation in exon 34 of *SRCAP* is a mandatory feature for a diagnosis of FHS. This is contrary to the report put forth by Le Goff et al. [14]. Six of their nine subjects were found to have mutations in exon 34 within the boundaries we describe, and they proposed that their three mutation-negative individuals indicate genetic heterogeneity for FHS. However, we reviewed the two photographs of their *SRCAP*-negative patients and did not believe their facial features were consistent with a diagnosis of FHS.

A high frequency of associated anomalies was seen in this study (33/52 had at least one major anomaly requiring medical intervention); however, none are pathognomonic for FHS. This large cohort of FHS individuals clarifies which clinical features are observed frequently and informs patient management guideline development. For example, celiac disease was initially thought to be more common in FHS, however, only 2 of 52 subjects had this finding. Although this is more than expected in comparison to the general population, the numbers are not such to suggest generalized screening. In comparison, genitourinary, ocular and dental issues were seen often enough to warrant investigations.

### Suggestions for management

Based on our clinical data, we suggest the following guidelines for the care of individuals with FHS:

1. Sequencing of *SRCAP* exons 31–34 in all suspected cases to confirm the diagnosis

2. Complete assessments of auditory and visual systems

3. Renal and urinary tract ultrasound

4. Neurologic assessment if there is a suspicion of seizures

5. Dental hygiene to prevent cavities and to monitor for malocclusion

6. Evaluation for growth hormone deficiency at baseline, to be repeated if loss of growth velocity occurs

7. Monitoring of bone age and pubertal timing. In cases of precocious puberty, referral to a pediatric endocrinologist

8. Psychoeducational assessments corrected for deficiencies in expressive language and sensory issues

9. Monitoring of behavioral disturbances and provision of early intervention

10. Counseling for families regarding recurrence risk (extremely low) and to offspring of individuals with FHS (50% chance).

## Conclusions

We have assembled the largest cohort of individuals with Floating-Harbor syndrome; documenting pathogenic mutations in *SRCAP* in 52 affected individuals. Characteristic clinical findings include short stature, delayed bone age, distinctive facial features, expressive language delay, and broad thumbs. If the characteristic facial gestalt is not present, the likelihood of finding a mutation in *SRCAP* is very low. It is not uncommon for an individual with FHS to have additional anomalies and health complications that require medical intervention and thus comprehensive baseline screening and surveillance is warranted. In general, individuals with FHS are healthy and despite some impairments, enjoy a good quality of life.

## Abbreviations

FHS: Floating-harbor syndrome; SRCAP: SNF2-related CBP activator protein; CBP: CREB-binding protein; SD: Standard deviation; OFC: Occipitofrontal circumference; GH: Growth hormone

## Competing interests

The authors declare that they have no competing interests.

## Authors’ contributions

SMN and AD were involved in design, acquisition and analysis of data, and drafting of the manuscript. SM was involved in design, acquisition and analysis of data, and made contributions to the draft of the manuscript. EB, HB, MC, OC, JH, UK, NM, SMW and MF were involved acquisition and analysis of data, and made contributions to the draft of the manuscript. RLH and DEB were involved in analysis of the data and made contributions to the draft of the manuscript. AA, BA, JEA, PB, TBO, FB, IC, BSC, LAD, AD, DF, FF, NG, GG, KH, YMCH, DH, AH, EMH, LHH, JI, CMJ, SGK, CAK, EPK, NVAM, DL, CL, IFML, LSL, FM, VM, JSM, STM, SM, DTP, KP, SP, AR, JS, JS, ELS, MEHS, AS, IKT, IvdB, BBAdV, JDWA, MLW, DW, JMW and CFOY were involved in acquisition of the data and made contributions to the draft of the manuscript. CMB and FORGE were involved in acquisition and co-ordination of the data. KMB was involved in design, analysis of data and critical revision of the manuscript. All authors read and approved the final manuscript.

## Supplementary Material

Additional file 1 Questionnaire used to collect clinical data. Click here for file

Additional file 2 **FSH-1.exon34.primers_10.11.11.xls (Primer pairs used to sequence exon 34 of *SRCAP*).** Click here for file
