# Supplementary material for: The phenotype of Floating-Harbor syndrome: clinical characterization of 52 individuals with mutations in exon 34 of SRCAP
Source: Orphanet J Rare Dis. 2013 Apr 27;8:63. doi: 10.1186/1750-1172-8-63 (PMC3659005; doi:10.1186/1750-1172-8-63)
Supplement: Additional file 1 — Questionnaire used to collect clinical data. [file 1750-1172-8-63-S1.doc]

FLOATING-HARBOR QUESTIONNAIRE

Patient

Sex

Ethnicity

Paternal age (years)

Gestation (weeks)

Birth weight (g)

Age at diagnosis

Age at last assessment (ALA)

Head circumference (cm) ALA

Weight (kg) ALA

Height (cm) ALA

Age at puberty

Pre-pubertal height

Bone age years versus chronological age

Triangular face

Distinctive nose

Low-hanging columella

Short philtrum

Thin upper vermilion

Wide mouth

Low set ears

Broad thumbs

Broad fingertips

Brachydactyly

Clinodactyly

Other skeletal

Dental issues

Gastrointestinal problems

Seizures

Other health issues/ /serious illnesses/hospitalizations

Behaviour issues

Attention Deficit/Hyperactivity

Psychiatric issues/Anxiety/Depression

High pitched voice

Speech delay

Intellectual development/Developmental Delay/Mental Retardation

*ALA – at last assessment
